# Long-term physical and psychological symptoms in Syrian men subjected to detention, conflict-related sexual violence and torture: cohort study of self-reported symptom evolution

**DOI:** 10.1016/j.eclinm.2023.102373

**Published:** 2023-12-14

**Authors:** Coleen Kivlahan, Mohammad AlSharif, Ingrid Elliott, Agustin Garcia Pereira, Zina Hallak, Reem Yonso, Ahmad Odaimi, Naser AlHafez, Mahmoud Aswad

**Affiliations:** a Department of Family Medicine, University of California San Francisco, USA; b Synergy for Justice, London, United Kingdom; c Insight Centre for Data Analytics, University of Galway, Galway, Ireland; d Lawyers and Doctors for Human Rights, https://ldhrights.org

**Keywords:** Syrian Arab Republic, Males, CRSV, Torture, Detention, SRH, FME, Physical and psychological symptoms

## Abstract

**Background:**

Since March 2011, more than 1 million people, mostly men, have been arrested, detained, and tortured by the Assad regime. Published literature does not reflect the evolution of symptoms after male sexual and physical violence in detention. This cohort study examines the constellation and evolution of self-reported symptoms after male conflict-related sexual violence (CRSV) in Syrian state detention.

**Methods:**

Sexual, psychological, and physical symptoms and conditions experienced by a cohort of 106 male detainees after CRSV in Syrian regime detention were evaluated over a ten-year period (2012–2022). Men sought forensic medical expert evaluations (FMEs) to document torture and later consented to semi-structured interviews (SSIs), a median of 8.8 years after their detention. A standard data collection tool was used to assess symptoms and conditions during FMEs (Time 3), and at the time of the SSI (Time 4), during which men also reported symptoms experienced during detention (Time 1) and after detention release (Time 2).

**Findings:**

30.2% of men spent more than 1 year in detention and 9.4% were detained >5 years. 90% reported being slapped, punched, kicked, hit with objects, 60.4% of men reported torture with multiple devices, and 48.1% reported being burned or electrocuted. Multiple sexual violence types were reported during detention: 97.2% forced nudity, 45.3% violence to genitals or anus, 30.2% collective sexual humiliation, and 9.4% rape. Men recalled nearly universal presence of acute pain, bleeding wounds, skin infections, sleep disturbances, fear, sadness, anxiety, and despair during detention. By Time 4, acute physical and psychological conditions were fading or absent, while scars, avoidance, intrusive memories, lack of trust, self-isolation, chronic pain, anger, and low self-esteem were reported by ≥50%. The most persistently reported symptoms following detention through the SSI included scars, pain, intrusive memories, and avoidance in ≥50% of men. At the SSI, 26.4% of men reported erectile dysfunction and 23.6% challenges with sexual relations.

**Interpretation:**

Men reported persistent symptoms and conditions years after CRSV, torture and detention. The unique constellation of findings and their evolution in male CRSV survivors, particularly increasing rates of anger, distrust, and self-isolation, must urgently inform design and delivery of support services and health care.

**Funding:**

This study was funded by the United Kingdom Foreign Commonwealth and Development Office and the 10.13039/501100000267Arts and Humanities Research Council through the project ‘Understanding and Addressing the Impact of Invisibility on Conflict-Related Male Sexual Violence in Syria'.

**Research in context**

**Evidence before this study**

While little is known about the prevalence of male sexual violence in conflict settings, even less is known about long-term symptoms and conditions suffered by male conflict-related sexual violence (CRSV) survivors. Prior studies reflect point prevalence of acute conditions; some studies describe survivor and stakeholder-reported effects at varying times following CRSV.

**Added value of this study**

This is the first study to our knowledge in which male CRSV survivors described physical and mental health symptoms over four time periods. Memories of detention and the post-release periods were described during the forensic medical evaluation (FME) and semistructured interview (SSI). The reported evolution of symptoms provides insight into conditions which persist, resolve or grow over time. Given the typically prolonged time to access forensic evaluations and services after detention, health care professionals’ understanding of these evolving symptoms is essential.

**Implications of all the available evidence**

Men subjected to CRSV while detained are at risk for medical and psychological symptoms lasting years. To achieve improved access to effective treatment, recognition of the constellation of symptoms unique to men (anger, loss of confidence, self-esteem, avoidance, and lack of trust) must be at the heart of access and care design. It is vital for informed policy to demand attuned services for men to improve livelihoods, family and community cohesion, and reduce conflict on many levels.

## Introduction

More than 12 years have passed since the Syrian revolution began. Starting with popular demonstrations against the Assad regime, the situation evolved into a “complex set of conflicts” including multiple State and non-State actors.^1^ In the absence of a comprehensive solution to the conflict, the Syrian people have suffered extraordinary consequences, with no end in sight. Since March 2011, an estimated 350,000–614,000 Syrians have died, over half the population has been displaced. More than 1 million were arrested, 154,000 Syrians remain in detention or were forcibly disappeared, and at least 15,000 died in detention at the hands of the regime's security services.^2^^,^^3^ While women and girls were also detained, the vast majority of those detained were men and boys.^4^ The lack of official figures hides the ongoing suffering and sequelae.^5^^,^^6^ The breadth of international crimes in Syria is extraordinary, including mass casualties, murder, torture, hostage-taking, enforced disappearance, CRSV, indiscriminate bombardment, use of chemical weapons, deprivation of food, and destruction of hospitals and schools.^7^^,^^8^

While there is limited literature about the prevalence of male CRSV during detention, there is even less regarding men's direct perceptions of their long-term symptoms and physical conditions affecting their well-being during and after detention.^9^, ^10^, ^11^ A Syrian NGO (Lawyers and Doctors for Human Rights, ‘LDHR’) documented physical and psychological evidence of torture against detained Syrian citizens from 2012 to the present, across multiple detention sites. LDHR's doctors, using the international guideline Istanbul Protocol,^12^ have performed more than 500 FMEs during active conflict. In human rights reports, LDHR documented the extraordinary rate of CRSV in male detainees.^13^ This data led us to investigate the evolution of self-reported symptoms and conditions in male Syrian detainees. To inform our research, we reviewed relevant studies on male Syrian refugees and detainees, other male CRSV survivor populations, male prisoners, and adult male survivors of childhood sexual abuse.

Studies of the Syrian conflict's effects on the male population offer insights into our study participants' specific challenges. Studies indicate high levels of psychological distress (60% prevalence of PTSD and depression) in Syrian men displaced to Iraq^14^; 80% of 24 Syrian torture and sexual violence survivors had PTSD (1998–2001)^15^; 34% PTSD in displaced married Syrians in Jordan.^16^ The All Survivors Project (ASP) key informant interviews reported shame, loss of confidence, sleep disorders, feelings of powerlessness, confusion, suicidal thoughts, feelings of emasculation, stigma and self-blame in Syrian males following sexual violence, as well as effects on spouses and families.^17^ Studies detailed acute and chronic physical conditions, such as headache, low back pain, and joint pain^14^^,^^15^ and broader effects such as impaired interpersonal and couple intimacy,^16^ and other social, economic and legal problems.^17^

Key studies from 2000 to 2022 on male CRSV typology and symptoms also informed this research.^18^, ^19^, ^20^, ^21^, ^22^, ^23^ Studies typically included single point-in-time interviews with male survivors and reported the occurrence of a wide range of sexual violence types. Men reported that physical torture often accompanied sexual torture.^18^ Across multiple studies of CRSV and political imprisonment, acute and chronic physical symptoms and conditions were reported, including traumatic genital injuries and pain, rectal fistulae, erectile dysfunction, and sexually transmitted infections. Acute mental health concerns included denial, memory loss, self-blame, shame, humiliation, sleep and eating disturbances, while chronic mental health concerns included chronic pain, dissociative reactions, compulsivity, lack of trust, reduced intimacy, and body image changes.^18^, ^19^, ^20^, ^21^ Multi-dimensional impact studies described short- and long-term mental and physical consequences, as well as economic, social, and familial effects.^19^ Beliefs about masculinity limited the ability to disclose traumas or report emotional pain, with male CRSV survivors interviewed years after detention remaining avoidant regarding their sexual trauma. They focused instead on immediate problems such as daily stressors, work and displacement, often describing their experiences as torture, not sexual violence.^18^, ^19^, ^20^, ^21^, ^22^, ^23^

Male prisoners are subjected to higher rates of sexual and physical violence than non-prisoners.^24^ Adult male survivors of childhood sexual violence report disrupted self-esteem, shame, worthlessness, and loneliness, and reported a 30-fold increased risk of suicide attempts as adults. In descriptive studies, male survivors reported numerous somatic symptoms such as headaches, gastrointestinal conditions, decreased appetite, and weight loss, as well as mood disturbances, anxiety, suicidal ideation and self-harm, substance use, sleep, and sexual difficulties.^25^ Qualitative, retrospective single point-in-time interviews of adult male sexual abuse survivors reflected challenges with sexuality, interrelationships, powerlessness, and stigmatization.^26^

Published literature describes acute and chronic psychological and physical health findings associated with sexual violence as reflected by survivors or service organizations at single points in time. There remains a major gap in the literature regarding male detainees' self-reported symptoms and conditions over time. The study's overall research aim was to investigate the evolution of survivor-reported symptoms and conditions following CRSV in detention. Study objectives include the assessment of the constellation of physical, sexual, and psychological symptoms and conditions during and after CRSV in detention as reported by Syrian men, change in reported symptoms and physical findings over time, and the description of persistently reported symptoms and findings over time. The primary hypothesis was that physical symptoms would fade, and chronic psychological symptoms that affected sexual relations and well-being in men would dominate in Time 4.

Using an observational cohort design, we evaluated a single cohort of male CRSV study participants' responses at two points in time: during their FME (Time 3) and the research interview (SSI, Time 4). Each of these interviews included structured questions about participants’ time in detention (Time 1) and the period after detention release (Time 2), as well as their current symptoms and conditions.

## Methods

This study used data extracted from FMEs conducted from 2016 to 2022, and SSIs completed from October 2021 to March 2022. To respond to the research aim, all study participants had an FME (Time 3) in which prior and current symptoms and conditions were assessed and forensic findings were documented, as well as a subsequent SSI (Time 4) over the nearly 10-year study period. Specifically, participants were asked to reflect on inflicted injuries and ill-treatment, as well as psychological symptoms and physical findings occurring during the periods of detention (Time 1) and post-release (Time 2). Thus, Time 1 and 2 data were constructed from retrospective recall of specific, self-reported memories from the detention and post-release periods during the interviews, and concurrent symptoms and findings were reported at Times 3 and 4. Consistent with international protocol,^12^ an FME includes an extensive recall of events in detention and the post-release impact. Mitigation of expected recall bias during the FME and SSI included open-ended questions with requests for detailed examples of key events.

Between 2012 and 2021, LDHR conducted 535 FMEs for Syrian men and women living in Syria and in neighbouring countries; 346 involved adult males who were detained and tortured. LDHR physicians conducted all FMEs using a standard format developed by Synergy for Justice and LDHR experts, informed by the Istanbul Protocol: Manual on the Effective Investigation and Documentation of Torture and Other Cruel, Inhuman or Degrading Treatment or Punishment.^12^ Physicians were trained and mentored in conducting FMEs in tortured, detained populations using survivor-centred approaches; typical duration of an FME is 3–5 hours. Upon request, survivors were referred for FMEs through confidential referral pathways from medical and other support service organisations. Further operational details are not published due to security concerns given the sensitivity of LDHR's work.

Study eligibility included male gender, FME completion, self-reported CRSV in Syrian detention during the study period, and current contact information. Based on international foundational definitions,^9^^,^^27^^,^^28^ eight sub-categories of sexual violence were utilised in this study. These included any form of rape as defined in the International Criminal Court's Elements of Crimes^29^; as well as violence against genitals including burning, electrocution and mutilation; collective humiliation with sexualised elements; forced witnessing of sexual violence; forced nudity; threats of sexual violence against self, others present or loved ones; and verbal sexualised abuse. Attempts were made to contact 145 men who met all study inclusion criteria. 106 men consented after study goals and informed consent were described (39 refused or were unable to be reached). Additional outreach was limited by extraordinary circumstances including active ongoing Syrian conflict, displacement, and migration.

All 106 study participants′ FMEs interviews and examinations were retrospectively reviewed, and data was extracted by five experienced physician-interviewers in advance of conducting SSIs. Participants had the option of in-person or remote interviews; 86% of the interviews were conducted remotely given the risks of travel within Syria and COVID-19 transmission. Remote interview concerns (safety, privacy, confidentiality) were addressed through training and escalation protocols. Interviews were conducted in Arabic, lasting 60–120 minutes, and were not recorded for security and confidentiality reasons. Themes and guiding questions for SSIs were developed based on the review of the literature as described above. Prior to the SSIs, physician-interviewers were trained on research protocols and use of open dialogue, building on information previously documented in the FME.

A dual language (English-Arabic) online data entry form (DEF) was created using the tool Paperform (Integrations|Paperform: Online Form Builder And Form Creator) to capture data from the FME and the SSI via a secure link. The DEF consisted of 66 quantitative questions (multiple-choice entries including lists of physical, psychological, and sexual reproductive health (SRH) conditions, numerical and date entries) and 105 qualitative open text data fields capturing free-form responses that augmented quantitative responses. Approximately half of all study data was extracted from FME data, and the remainder through the SSI. Quality and consistency checks were performed on the first 5 DEFs for each physician-interviewer, accompanied by team meetings to identify interview challenges, data review, mentoring, and coaching. Self-care sessions for physician-interviewers were held throughout the SSI phase.

### Statistical analysis

From the literature review and expertise documenting Syrian international crimes in detention, we identified five research foci: key demographic data and circumstances of arrest; typology and circumstances of ill-treatment; reported symptoms and physical findings over time; social, economic, legal, and radiating effects; and access to support services. This study describes results from the first three foci.

Data was exported to tabular format using Paperform's built-in automated function. Data pre-processing isolated quantitative from qualitative data. Due to the limited interview time (60–120 min) and the commitment to respond to study participants' unique experiences, some data entry points were not completed for some study participants. Missing data values were coded using a “no-data” category since the sample size was insufficient to implement other strategies. For data with multiple, predefined quantitative responses (e.g., lists of physical, psychological and SRH conditions), categorical binary variables indicated presence or absence of each sign, symptom, and type of CRSV or violence. The resulting dataset was imported into the open-source business intelligence tool “Metabase” (https://www.metabase.com/) for visualisation and analysis. An agile development process was followed to build multiple data dashboards, with requirements addressed and validated with researchers.

As described above, the DEF captured quantitative data on key study outcomes such as CRSV typology, other forms of violence, and physical, psychological and SRH findings, as well as qualitative data to augment quantitative responses. Translation of free-text Arabic qualitative responses was performed to ensure creation of dual language responses, then uploaded into Nvivo R.17 for thematic analysis. For the purposes of this study, qualitative data in the DEF was reviewed to supplement and verify quantitative responses for key study outcomes. A separate inductive analysis and open coding of the complete qualitative data is underway by NUIG social scientists to consider qualitative research questions. An expert consensus process was conducted by two authors with extensive CRSV and forensic clinical documentation experience. Categorization of qualitative responses into pre-defined quantitative categories (CRSV typologies, other forms of violence, and physical, psychological and SRH conditions used for DEF quantitative categories) was performed. A tabular binary grid including forms of violence and a time-based grid for symptoms and findings were developed. Expert clinical review of physical, psychological and SRH findings combined similar constructs based on congruence of clinical content. Intervals between study periods (T1-T4) were calculated from DEF entry points using the DATDIFF (days) Excel function. Analyses were performed using a combination of Microsoft Excel for handling and preparation of tabular data, and a custom python script for statistical analysis using pandas and SciPy (stats module) python libraries. The two tools were used together with Metabase for creating visualisations, in this case, including the python library Plotly.

Four main analyses are presented. First, using descriptive statistics, we provided the mean, median, range and prevalence of all variables, including demographics, circumstances of arrest and detention, types of violence and the presence of symptoms and conditions. We investigated the evolution of the prevalence of symptoms and conditions within each time period and over the full study period. To describe the trajectory of the most prevalent symptoms and conditions over time, all 121 physical, psychological and SRH symptoms were filtered to remove those with a prevalence of *<*10% at all four study times (n = 45) and those with identified concerns regarding metric validity (due to DEF category labels, researcher bias, or participant self-report bias (n = 12)). A multivariate analysis was also considered during the statistical analysis plan, but since multivariate analysis requires multiple data points to produce reliable and valid results (and to avoid overfitting or underfitting the model), it was not pursued due to the relatively small number of study participants and extensive number of variables under consideration.

Attention to trauma, confidentiality, and interview safety centred the research. Physician-interviewers employed trauma-informed interview techniques compliant with the Murad Code (www.muradcode.com; Draft Version 2020, Working Version 2022), escalation protocols, case management debriefs, and confidential referrals to support services. Self-care protocols were in place to limit trauma exposure and assure welfare. Ensuring the confidentiality of participants and safety of data were of paramount concern; LDHR's confidentiality and data management security protocols for FMEs were applied to this project. Study participants were assigned a unique case code; no identifying details were entered into encrypted research databases maintained in a limited access safe/vault. Password-controlled, encrypted communications technology was security-checked and risk-assessed. Interview safety measures included risk assessments, safe and restricted access sites, and access to emergency response. A rigorous, three-step informed consent (IC) process was implemented. LDHR case managers first contacted eligible participants to offer referral support for acute needs, confirm updated contact information and identify men with interest in ongoing engagement (N = 145). Physician interviewers contacted those interested in opportunities for research and provided research purpose, requirements, risks and benefits; then interested participants were recontacted for full informed consent, identifying 106 study participants.

### Ethics statement

The study activities were approved by the institutional review board of the National University of Ireland, Galway's (NUIG) Research Ethics Committee (REC) which granted ethical approval on 5th July 2021 (REC Application Reference Number: 2021.05.009). We obtained written informed consent for the use of research data, as approved by the NUIG REC.

### Role of the funding source

Funders of this study, the United Kingdom UK Foreign Commonwealth and Development Office and the Arts and Humanities Research Council had no role in study design, collection, analysis, or interpretation of data, writing or in the decision to submit the paper for publication. Four authors had access to the full dataset (Kivlahan, Elliott, AlSharif, Pereira) and all authors made the decision to submit for publication.

## Results

Study participant demographic and detention information is summarised in Table 1. Participants, detained throughout Syria, were mostly married, childless, Muslim, Arab Sunni men with high school education or beyond and were stably housed.

**Table 1:** Study participant demographic and detention information.

	Count	Percentage (n = 106)
**Ethnicity/race**		
Arab	105	99.1
Kurd	1	0.9
**Religion**		
Muslim	106	100.0
**Residence at time of arrest/detention**		
Aleppo	16	15.1
Damascus	17	16.0
Hama	15	14.2
Homs	6	5.7
Idlib	29	27.4
Lattakia	13	12.2
Rif Damascus (surrounding Damascus)	9	8.5
Living outside Syria, arrested at checkpoint	1	0.9
**Level of education at time of arrest**		
None or primary school	47	44.3
Secondary school/high school	22	20.8
University studies or beyond	37	34.9
**Marital status at time of arrest**		
Divorced	2	1.9
Married	63	59.4
Single	41	38.7
**Number of children**		
0	42	39.6
1-2 children	19	17.9
≥3 children	36	34.0
Missing data	9	8.5
**Displacement status prior to arrest**		
Displaced	5	4.7
Homeowner	39	36.8
Living with parents or family	42	39.6
Renting	17	16.0
Missing data	3	2.8

All study participants were detained at least once, for variable lengths of time. The median age at the time of their first arrest/detention was 28 years old (range: 15–55). Almost all (91.5%) were detained in military intelligence sites, while 63% reported being detained in additional regime detention sites. Only six men were held by a non-governmental or foreign group; the remainder were held by Assad regime forces. During detention, almost all men faced interrogations conducted by guards or militia (98%), while 58.5% reported interrogations by middle-ranking officers.

**Fig. 1:**
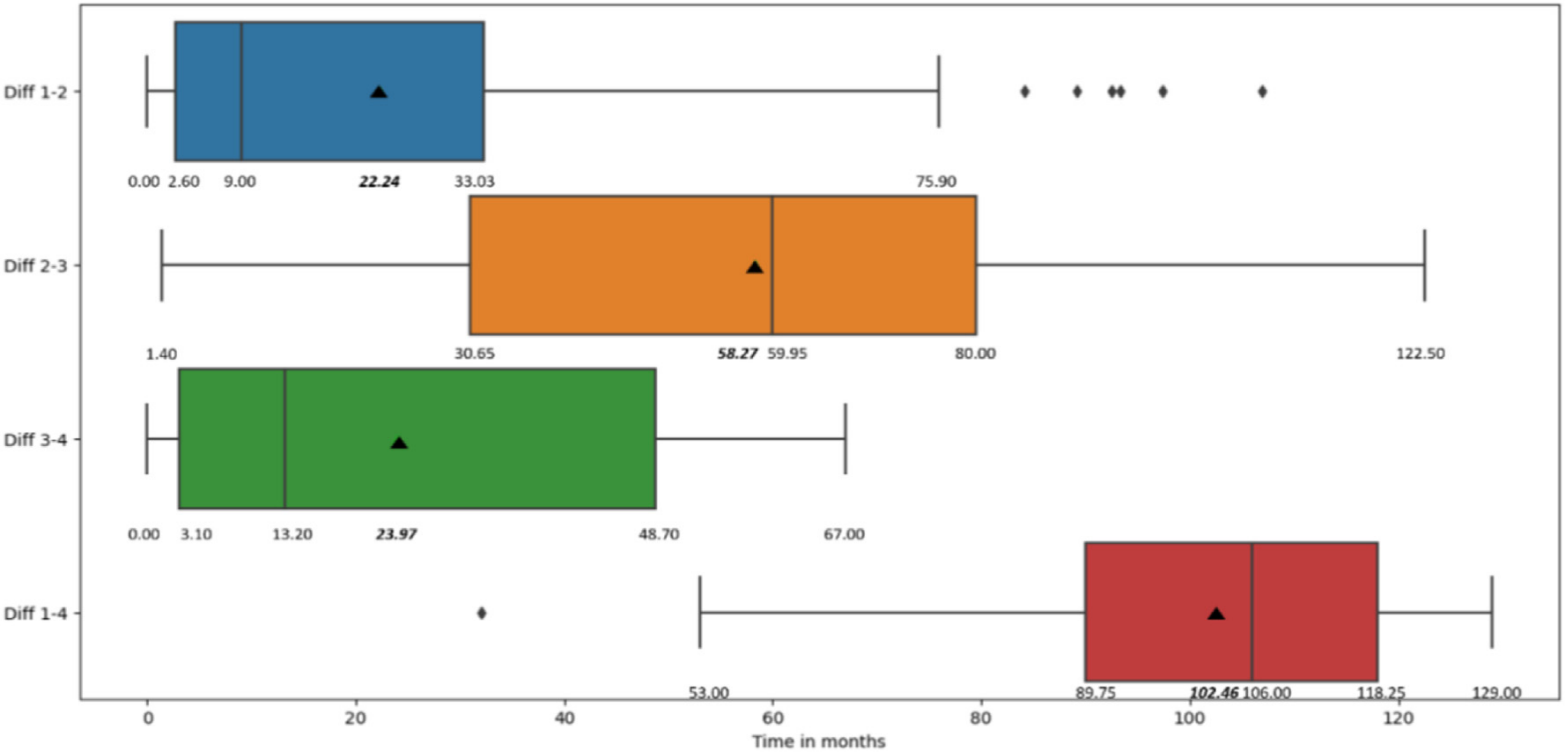
Intervals between study times 1–4. Box plots reflect the variation in study participants' duration of detention, post-release, FME and SSI timeframes. X axis is time in months; Y axis includes four time intervals: Diff 1–2 (time from detention to post-release period); Diff 2–3 (time from post-release period to FME); Diff 3–4 (time from FME to SSI) and Diff 1–4 (time from detention to SSI). The minimum and maximum, quartiles, median (vertical line in the box), mean (black triangle in the box), and outliers (black diamonds) are depicted for each study time interval in months.

Four unique periods were evaluated for each study participant. Time 1 and 2 data were based on retrospective recall of memories from participants' detention (Time 1) and post-release periods (Time 2); concurrent symptoms and findings were captured during the FME (Time 3) and SSI interviews (Time 4). Study participants reported a median of 9 months between detention and post-release periods (Fig. 1, Diff 1–2). The FME occurred at a median of 58.3 months after the post-release period (Diff 2–3) and a median of 13.2 months elapsed until the SSI (Diff 3–4). Study participants′ median age at the time of the SSI was 38 years (range 22–62), ten years older than at their detention.

Overall, the median study period from Time 1 to 4 was 106 months (range 53–129 months; Diff 1–4).

Detention experiences varied by duration, number of sites and occurrences. The median number of detentions during the study period was 1.0, mean of 1.4 (range 1–11). The mean duration of detention was 18.9 months (measured by single detention or the longest detention if participants reported multiple detentions), median was 8 months (range 2 days–119 months) and the longest duration was almost 10 years. The distribution of detention duration included a few men who were detained for less than a month and about half who were detained under 1 year. Notably, almost a third (30.2%) suffered extensive detentions between 1 and 5 years and 9.4% spent more than 5 years detained. Most men were detained multiple times in multiple sites; 40% were detained in 5–8 sites, and 8.6% reported >8 detention sites.

**Fig. 2:**
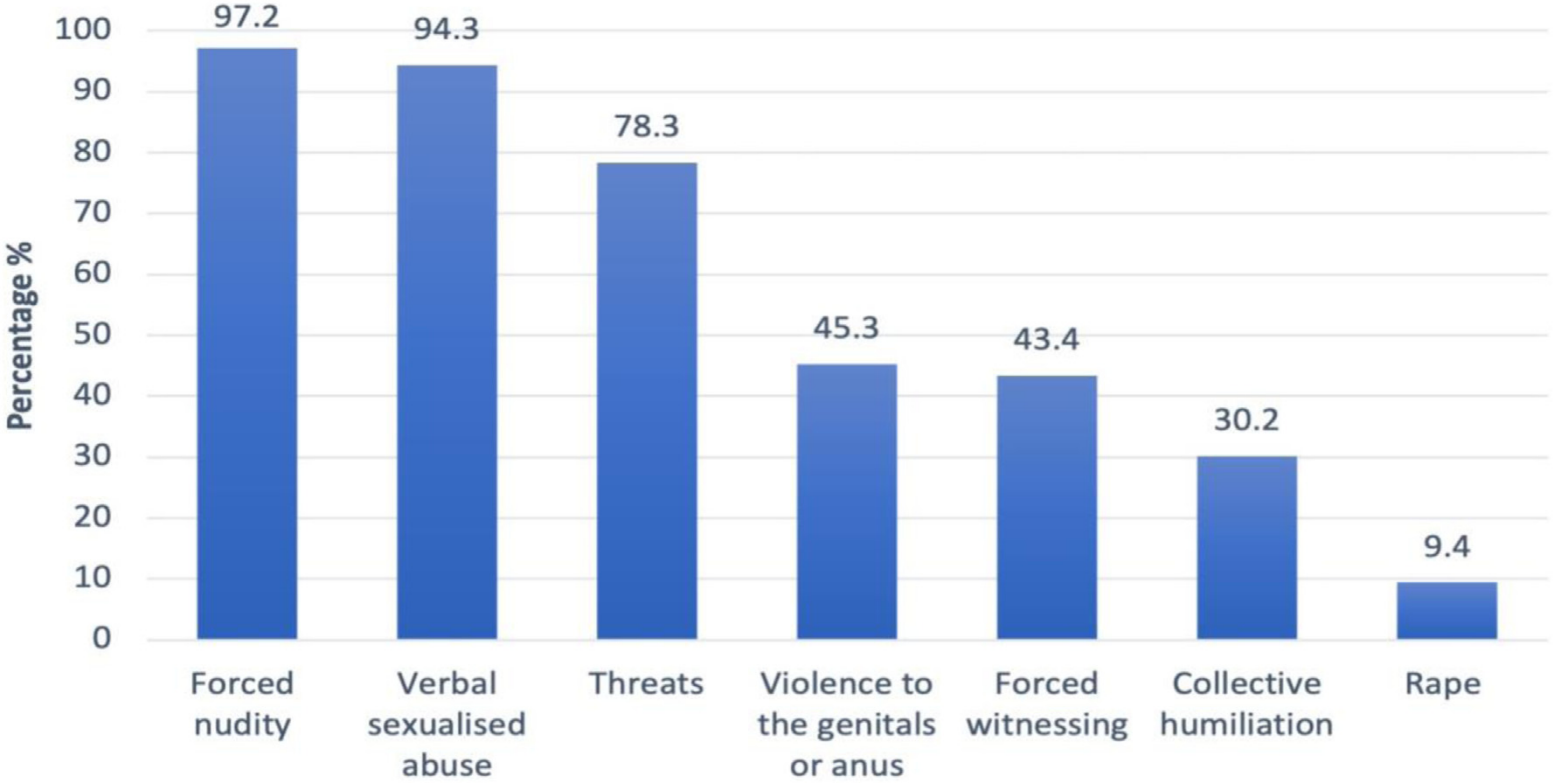
Prevalence of types of reported CRSV during detention. X axis represents the most prevalent types of conflict related sexual violence. Y axis is percent prevalence reported.

Study participants reported that CRSV occurred most often during the first few days of detention. Detainees reported abuse by an average of 5 perpetrators, most of whom were lower-ranked guards and jailors. Multiple CRSV types experienced during detention were reported (Fig. 2). Forced nudity, reported by virtually every study participant (97.2%) occurred at a variety of times: 92.4% during arrival searches, 69.8% during interrogations or torture, and 54.7% while in their cells. Most men reported verbal sexualized abuse and threats. Sexual threats had varying foci for example: 58.5% reported threats of sexual violence toward female loved ones, 70.7% threats against themselves, 34.9% threatened with rape, and 38.7% with sterilisation or castration. In addition, all men who reported experiencing verbal sexualised abuse described at least one other form of sexual violence simultaneously being inflicted upon them. 45.3% of men reported direct violence to their genitals or anus such as genital beating (39.4%) and 13.2% genital electrocution. 43.4% of men reported being forced to witness sexual violence. 9.4% reported anal penetration.

Study participants reported multiple concurrent episodes of CRSV, physical trauma, and psychological abuse while detained. Most men reported the use of ligatures/restraints (81.1%) or blindfolds (75.5%), more than 90% reported being slapped, punched, kicked, hit with objects, and 100% faced verbal insults. 60.4% of men reported experiencing a variety of torture devices, and 48.1% reported being burned or electrocuted on some body part(s). Most men reported restraint of movement and forced positions (78%), and 80% reported suspension.

Reported symptoms and conditions were analysed by prevalence within each study time, change in prevalence across study time periods, and symptom persistence over time. Within each study time, the ten most prevalent symptoms and conditions are described in Table 2. During Time 1 (detention), participants reported nearly universal acute symptoms such as body pain in multiple sites, bleeding, open wounds, and skin infections, as well as acute psychological symptoms including highly prevalent reports of shame, fear, sleep disturbance, sadness, and anxiety.

**Table 2:** The ten most prevalent reported symptoms and conditions at each study time. All symptoms and conditions were analyzed by Time 1–4; the ten symptoms and conditions with the highest prevalence are noted.

Time 1	Body Pain (100%), Bleeding/Wounds (96.2%), Shame (96.2%), Insomnia/sleep disturbance (94.3%), Bruising (93.4%), Prolonged or intense fear (89.6%), Sadness (88.7%), Anxiety (87.7%), Skin conditions (84.0%), Despair (84.0%)
Time 2	Body Pain (94.3%), Insomnia/sleep disturbance (91.5%), Intrusive memories (88.7%), Nightmares (87.7%), Scars (84.9%), Sadness (83.9%), Anxiety (83.0%), Prolonged or intense fear (77.4%), Avoidance (77.4%), Weakness (76.4%)
Time 3	Scars (86.8%), Intrusive memories (80.2%), Avoidance (79.2%), Body pain (78.3%), Insomnia/sleep disturbance (66.0%), Anxiety (64.1%), Nightmares (62.3%), Sadness (59.4%), Self-isolation (58%), Anger (57.5%)
Time 4	Scars (71.7%), Avoidance (71.7%), Intrusive memories (68.9%), Lack of trust (66.9%), Self-isolation (65%), Body pain (64.2%), Anger (57.5%), Low self-esteem (50.0%), Sadness (48.1%), Nightmares (45.3%)

In Time 2, the period following detention, more than 80% of all study participants continued to report body pain, sleep disturbance, sadness, anxiety, and fear. New onset of, or increases in, reports of intrusive memories, nightmares, scars, avoidance, and weakness appeared among the ten most prevalent reported symptoms during the post-detention phase. By Time 3 (at the FME), scars, intrusive memories, avoidance, pain, sleep disturbance, anxiety, nightmares, and sadness were still highly prevalent, with new onset of, or increase in, anger and self-isolation. At Time 4 (the SSI), scars, avoidance, intrusive memories, pain, anger, sadness, and self-isolation continued to be reported at high rates, while lower than in Time 3. Disturbing new symptoms emerged in Time 4, including lack of trust and low self-esteem, and self-isolation increased. Even years after detention, nightmares impacted 45.3% of study participants.

**Fig. 3:**
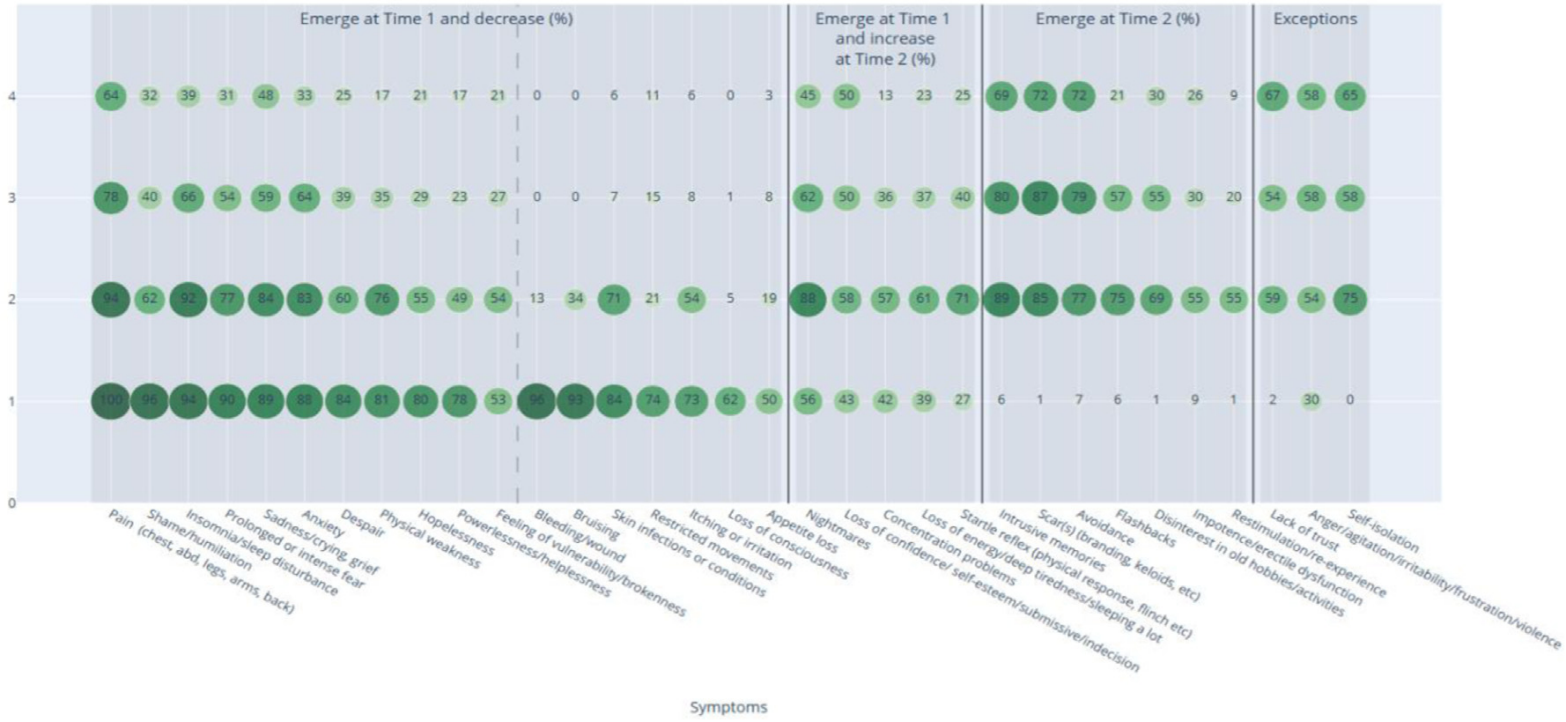
Evolution of high prevalence reported symptoms and conditions (%) over time. Symptoms and conditions with prevalence ≥50% in Time 1 or 2 are depicted on the X axis; % prevalence at Time 1–4 is noted on the Y axis shaded by level of prevalence. For example, on the left of Fig. 3, body pain is reported by 100% of men in Time 1 and decreases to 64% in Time 4. Prevalence of all symptoms/conditions is described in Supplementary Table S1.

To demonstrate changes in the evolution of symptoms and conditions reported over time, patterns of symptom prevalence are shown using a heatmap chart for 32 symptoms with a reported rate ≥50% of onset in Time 1 or 2 (Fig. 3). Lower prevalence symptoms and conditions are summarised in the text; a table reporting prevalence of all symptoms is appended as Supplement Table S1.

Three key patterns in symptom evolution emerged from analyses of Time 1–4. The first pattern includes symptoms/conditions which emerged in Time 1 and slowly decreased (improved) over time, depicted on the left side of Fig. 3. Importantly, seven high prevalence symptoms which emerged during detention (pain, shame, sleep disturbance, fear, sadness, anxiety, and despair) were still present in ≥25% of men at Time 4. Highly prevalent psychological symptoms in Time 1 including weakness, hopelessness, and powerlessness decreased to <25% of men by Time 4. Some acute symptoms which emerged in Time 1 were negligible by Time 3 and Time 4, as expected, such as bleeding, skin infections, bruising, itching, restricted movement, loss of consciousness and appetite loss (left side of Fig. 3). Lower prevalence symptoms that began in detention and improved over time included loss of spatial/temporal awareness, dissociative reactions, breathing difficulties and panic, incontinence and painful urination, headaches and chest pain, broken teeth/bones, constipation, vision, and gastrointestinal conditions.

The second pattern (middle of Fig. 3) includes symptoms/conditions which emerged in detention (Time 1 reported rates ≥25%) increased (worsened) at Time 2, and by Time 4 persisted in ≥25% of men, including nightmares, loss of confidence, and startle reactions. Lower prevalence symptoms with the same pattern included weight loss, low sexual desire, impact on sexual thoughts and relations, thoughts of revenge, memory problems and guilt/self-blame.

The third pattern (right side of Fig. 3) includes symptoms which were virtually absent during detention and emerged in Time 2; most of which slowly decreased from Time 2 to Time 4. Intrusive memories, scars, and avoidance decreased but were still reported with a high prevalence at Time 4 (69–72%), while 26–30% of men still reported impotence/erectile dysfunction and disinterest in prior activities at Time 4. Flashbacks and re-experiencing trauma continued to improve over time, as did lower prevalence symptoms such as reports of joining up to fight and blame of others.

Exceptions to the above patterns are notable: anger and lack of trust were the only symptoms that increased (worsened) from their onset in Time 1–2 to Time 4. Self-isolation worsened from Time 3 to Time 4 (far-right column, Fig. 3) and lower prevalence symptoms such as negative thoughts and mood were also reported more often in Time 4. While relatively low-prevalence, suicidal thoughts and self-harm are important clinical markers of distress and in our study, most prominent during detention. Reported suicidal thoughts and actions dropped from 15.1% during detention to 8.5% at Time 4. Twelve percent of men reported self-harm during detention which decreased to 1.9% at Time 4. Tinnitus/hearing difficulty and nerve impairments were also low prevalence symptoms which persisted over time, as expected based on the chronic nature of those conditions.

**Fig. 4:**
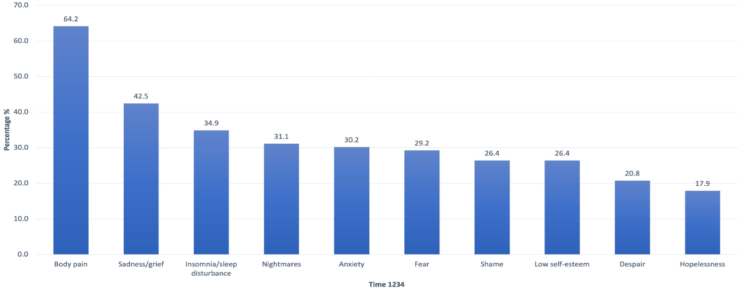
Prevalence of men reporting persistent symptoms across T1–T4 (top ten symptoms). X axis includes the ten most prevalent symptoms reported by a subgroup of men who reported these symptoms across all four time periods. Y axis depicts the percent prevalence across Time 1–4, for example 64% of all study participants reported pain during detention and all subsequent study periods.

In addition to analysing the changing prevalence of the most reported symptoms across study periods, some men reported the same symptoms and conditions (symptom persistence) over all four time periods. Subgroup analyses found that 64.2% of men reported body pain persistently over all four time periods (Fig. 4). Additionally, sadness, sleep disturbance, nightmares, and anxiety persisted over time in 30.2–42.5% of men. Importantly, 17.9–29.2% reported persistent fear, shame, low self-esteem, despair, and hopelessness.

**Fig. 5:**
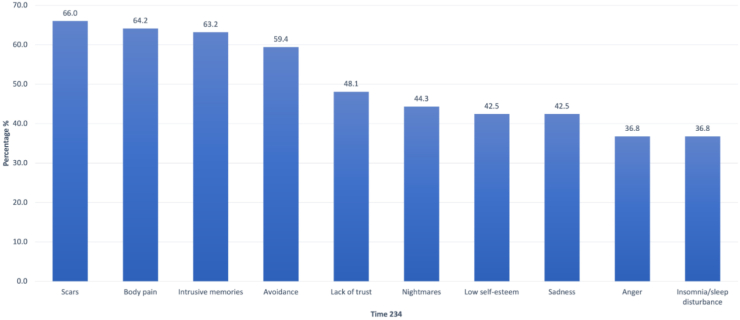
Prevalence of men reporting persistent symptoms emerging after detention, T2-4 (top ten symptoms). X axis includes the ten most prevalent symptoms reported by a subgroup of men who reported these symptoms beginning after detention and extending through the SSI. Y axis depicts the percent prevalence across Time 2–4, for example 63.2% of all study participants reported intrusive memories after detention and all subsequent study periods.

As described in Fig. 3, new and important symptoms emerged following detention. Fig. 5 shows the prevalence of men consistently reporting the same symptoms emerging after detention through the SSI (Times 2–4). Four symptoms are reported in >50% of men, including scars (66.0%), body pain (64.2%), intrusive memories (63.2%), and avoidance (59.4%). One third to nearly one half of men reported persistent anger, insomnia, low self-esteem, sadness, nightmares, and lack of trust.

While it was outside the scope of the initial research questions and not among the ten most prevalent symptoms reported by our study population, at the SSI, men reported sexual and reproductive health (SRH) symptoms such erectile dysfunction (26.4%) and challenges with sexual relations (23.6%). Preliminary analyses evaluated associations between the types of violence men reported and the development of SRH conditions (Supplementary Tables S2 and S3). Better understanding the relationships between types of violence and SRH conditions can guide the development of humanitarian and community health responses to improve gender-sensitive access and disclosure points while reducing cultural taboos and stigma for men. The initial findings warrant further investigation to identify other potential confounding factors in detention and conflict settings.

This paper's focus is on long-term physical, sexual and psychological symptoms. Additional study questions included self-reported family and work/economic impact and support service access. At Time 4, 70.2% of the men were displaced from their home and community, with almost half (48%) having fled government-controlled territories within days of their detention release. 79.8% reported lost work/income. 84.6% of interviewees reported that they feared arrest and were currently sought by the Syrian government.

More than 70% of the men reported sources of strength including faith, religion, and family. Ability to tell their story in their own words (34%) and moving to a new place (29%) were reported as helpful sources of support. 71% reported that they lacked knowledge of support services, and most (58.6%) reported that they were embarrassed to seek services. Only 5.7% (6 men) reported no residual physical symptoms and only one reported no psychological symptoms during the study interview (Time 4). Very few men described accessing formal support services as a major source of strength (3%), most identified key barriers of trust, pride, and distance from resources during humanitarian crises.

## Discussion

In the current study, we examined the evolution of symptoms and conditions in Syrian men following detention, torture and CRSV as reported during FMEs and SSIs. Self-reported symptoms and conditions changed between detention and that of the research interview (SSI), a median of 8.8 years later. The extreme and pervasive levels of violence and brutality in Syrian detention centres are clear from our findings; all study participants experienced multiple forms of physical, psychological, and sexual violence in detention, many endured multiple and prolonged detentions and interrogations in multiple sites. Men reported that CRSV began early in detention, and formed part of a broad pattern of violence, torture, and life-threatening conditions.

Given the reported levels of violence, the constellation of conditions during detention such as pain, bleeding/wounds, bruising, skin conditions, sleep disturbance, prolonged and intense fear, shame, sadness, anxiety, and despair is not surprising. As clinically expected, acute wounds, restricted movement, and loss of consciousness and appetite prominent during detention, were reported far less frequently during the periods after release. By the time of their FMEs, most physical symptoms/conditions were healed, but intrusive memories, avoidance and anger dominated men's concerns, and the time of the SSI, lack of trust, sadness, self-isolation, and nightmares augmented the reported impact on men's daily lives. Acute trauma has been reported to evolve into chronic pain syndromes and deep psychological symptoms known to affect well-being.^20^ By the time of the SSI, half or more of study participants reported physical scars, avoidance, intrusive memories, lack of trust, self-isolation, pain, anger, and low self-esteem. In addition, men reported self-harm and suicidal thoughts to the physician-interviewers (according to the experience of Syrian physicians performing FMEs, these taboo subjects in Syrian culture and religion are rarely discussed in any setting).

This study's self-reported long-term symptoms and conditions following CRSV build on previously cited work of male sexual violence from aid agency and stakeholder reports, FME data extraction and several studies evaluating effects distant to the events.^18^, ^19^, ^20^, ^21^, ^22^, ^23^, ^24^ While the All Survivors Project (ASP)^20^ reported that Syrian males described many similar symptoms including shame, loss of confidence, sleep disorders, feelings of powerlessness, confusion, suicidal thoughts, feelings of emasculation, stigma and self-blame, the study was conducted with a single SSI with 66 key informants, and not with survivors. Gray^30^ applied data from one-time interviews (individual and groups in Ugandan refugees) to focus on sense-making after CRSV, reporting many similar themes such as isolation, powerlessness, and sexual relations concerns. To our knowledge, no other published study describes survivor self-reported symptoms at different periods in time over nearly a decade of active conflict.

The policy implications of these results are widespread and include impact on male survivors of CRSV and detention, their families and the resulting risks of intergenerational trauma, economic stability and community cohesion, as well as peace and nation-building. Generations of angry, distrustful, isolated men present risks to their own health including substance use, greater risk of cardiovascular disease, dementia, stroke, depression, anxiety, and premature death.^31^ For each man who experiences torture in detention, there are partners, children, families, and communities who suffer. The implications for service and health care practitioners are urgent: men are already reluctant to describe themselves as CRSV survivors and seek care, it is imperative that we train clinicians to recognize the unique ways that men adapt to sexual trauma over time. Research implications include the need for a better understanding of the well-being of families, the association between forms of male and female violence and SRH conditions, long-term impact on social cohesion and economic security, and strategies for improving earlier outcomes-driven support and interventions for men. This study aims to raise awareness about the evolving and persistent symptoms experienced by men detained and tortured.

There are several limitations to our study. Data are derived from a retrospective cohort of detained men within the context of the Syrian conflict; by nature of its design, this analysis does not represent all male survivors of CRSV, nor all conflict settings. Study participants and physician-interviewers were impacted by multiple contextual issues during the study period including ongoing conflict in Syria, displacement, and the Covid pandemic. In addition, men who volunteered to document their detention and torture for the purposes of accountability for international crimes, remained in contact with LDHR, chose to participate in this research, and lived mostly in northwest Syria may have acute or chronic symptoms different from other detainees. Yet in the safe, confidential setting of the FME and SSI, men spoke about culturally taboo subjects as well as masculine gender norms. Physician-interviewer biases, such as personal and clinical experience and internalised barriers to discussing sexual impact, may have affected interpretation of trauma histories, resulting in underestimation of findings and the actual scale of the violence.

Given this study's research aims, the optimal study design is a comparison of detained men with and without CRSV exposure, as well as direct interviews at multiple times to reduce the impact of memory distortion and suppression. One of the few relevant cross-sectional studies (compares male and female refugees in Germany with and without lifetime sexual violence exposure) found that 36.7% reported direct or witnessed sexual violence (32.6% of men; 45.9% of women). Those reporting sexual violence had higher rates of depression and PTSD symptoms compared with those without sexual violence histories, but the study did not evaluate physical findings, SRH symptoms or assess participants at multiple points in time. Study authors noted that “longitudinal studies should be conducted to investigate and understand the trajectories of trauma-related mental health outcomes in refugees.”^32^ In the context of this study, thousands of Syrian men who left detention were displaced, impoverished, and had no access to services; a comparison group of men with no CRSV exposure was impossible to identify during active conflict.

It is important to note that men detained early in the Syrian conflict (2011–2013) were often unable to access an FME for several years after detention due to limited availability of experts and human rights education (median Time 2–3 interval was 59.9 months, mean 58.3 months, IQR 30.56–80, and range 1.4–122.5). The study mitigated recall bias risk by use of open-ended questions with requests for specific details and examples of key events and clarifications on the timeframe of detention and post-release periods. Trauma recall over time was also likely affected by ongoing displacement, conflict, and lack of treatment access. During active conflict, these factors are not controllable. Despite these limitations, the prevalence of acute symptoms such as wounds, or long-lasting symptoms such as avoidance, changed in expected ways over time (wounds decreased, avoidance increased). Voluntary involvement of participants and physician-interviewers, clear study inclusion criteria, reported safety levels from physician-interviewers and participants, mentoring sessions, use of Arabic for all responses and standard data collection, and deployment of physician-interviewers with a decade of conflict-related experience in forensic evaluations of torture survivors reduced the risk of systematic biases.

We conclude by noting that, to our knowledge, this study is the first to describe self-reported physical and psychological symptoms and conditions in detained male CRSV survivors over time. Syrian men describe the evolution of their acute wounds to chronic pain; and the fear and despair they reported during and after detention morphs into avoidance, anger, isolation, and distrust. Documentation of these highly prevalent long-term symptoms catalyses the mandate for action to improve identification, early outreach, and treatment of men to limit the devastating sequelae of sexual and physical violence reported in this study. Our findings are particularly pertinent for countries in conflict or under repressive regimes where detention, CRSV and torture are common. In the words of one of the Syrian physician-interviewers, “The release of the survivors is not the end of their suffering but may just be the beginning of it.”

## Contributors

Kivlahan, C (design and conceptualisation, methodology, supervision, data analysis, writing original draft, review and editing paper), AlSharif M (design and conceptualisation, supervision, data acquisition and interpretation, drafting and editing paper, resources).

Elliott, I (supervision, design and conceptualisation, methodology, data curation, data analysis, validation, writing original draft, review and editing paper, resources), Garcia Pereira, A (formal data analyses, data curation, validation, writing original draft, review and editing paper, visualization), Hallak, Z (design and conceptualisation, data collection, review and editing paper), Yonso, R (design and conceptualisation, data collection, review and editing paper), AlHafez, N (design and conceptualisation, data collection, review and editing paper), Odaimi, A (design and conceptualisation, data collection, review and editing paper), Aswad, M (supervision, conceptualisation, review and editing paper, resources).

Administrative, technical, or material support: MA, IE, MAS.

Final approval of the version to be published: All authors reviewed and approved.

Accessed and verified all underlying data and responsible for the decision to submit the manuscript: CK, IE, MAS and AGP.

## Data sharing statement

Due to the sensitivity of collecting person-specific data on torture and detention locations noted during armed conflict, individual participant data will not be released. A data dictionary is available in the form of a blank DEF. Upon request, study protocols and informed description and forms are available. This manuscript was written in accordance with The Strengthening the Reporting of Observational Studies in Epidemiology (STROBE) standards.^33^

## Declaration of interests

Authors Kivlahan and Elliott serve as medical and legal expert consultants to Synergy for Justice and as researchers for Synergy for Justice and the funding entity UK Foreign Commonwealth and Development Office and the Arts and Humanities Research Council. Elliott also serves as a Deployable Civilian Expert for UK Foreign Commonwealth and Development Office, working with LDHR from 2012 to 2015 as a UK expert. Author Garcia-Pereira served as a paid consultant to LDHR for data analyses. Authors AlSharif, Halak, Yonso, AlHafez, and Odaimi consultants to LDHR. Author Aswad serves as executive director (a paid employee of LDHR), and in that capacity, manages other LDHR grants not directly relevant to the submitted work and accepts only travel expenses for his attendance at human rights meetings. There are no other conflicts to disclose.
