# Supplementary material for: Long-term physical and psychological symptoms in Syrian men subjected to detention, conflict-related sexual violence and torture: cohort study of self-reported symptom evolution
Source: eClinicalMedicine. 2023 Dec 14;67:102373. doi: 10.1016/j.eclinm.2023.102373 (PMC10770421; doi:10.1016/j.eclinm.2023.102373)
Supplement: Supplementary Materials Tables S1–S3 [file mmc1.pdf]

Appendix A. Supplementary Materials

**SUPPLEMENTARY TABLE 1.**<br>**Symptom and Condition Prevalence over Time 1-4**				
**Symptoms/Conditions**	**T1**	**T2**	**T3**	**T4**
Abnormality/injury to penis	3.77	9.43	2.83	1.89
Abscess	32.08	10.38	4.72	1.89
Anger/agitation/irritability/frustration	30.19	53.77	57.55	57.55
Anxiety	87.74	83.02	64.15	33.02
Appetite loss	50.00	18.87	8.49	2.83
Attachment to perpetrator/Stockholm Syndrome	0.00	0.94	0.94	0.94
Avoidance	6.60	77.36	79.25	71.70
Blame others	0.94	3.77	4.72	12.26
Bleeding/wound	96.23	13.21	0.00	0.00
Blood in urine	3.77	1.89	0.94	0.94
Breathing difficulties/Shortness of breath	30.19	14.15	6.60	8.49
Broken bones/fractures	18.87	16.04	8.49	4.72
Broken or lost tooth /dental problems	27.36	27.36	19.81	13.21
Bruising	93.40	33.96	0.00	0.00
Burns/burn scar	39.62	24.53	17.92	17.92
Chest pains	22.64	20.75	13.21	10.38
Colon cramps	0.00	0.00	0.00	0.94
Concentration problems	42.45	56.60	35.85	13.21
Confusion	0.00	0.00	1.89	1.89
Constipation	15.09	8.49	2.83	0.94
Cysts	0.94	1.89	0.94	1.89
Dehydration	15.09	1.89	0.00	0.00
Despair	83.96	60.38	38.68	25.47
Development of phobias	2.83	9.43	6.60	1.89
Difficulty walking/gait abnormality	35.85	24.53	11.32	9.43
Disassociation/dissociative fugue	19.81	9.43	7.55	2.83
Disfiguration/mutilation/disability	6.60	3.77	6.60	6.60
Disinterest in old hobbies/activities	0.94	68.87	54.72	30.19
Dizziness	1.89	3.77	1.89	0.94
Feeling of vulnerability/brokenness	52.83	53.77	27.36	20.75
Fever/sweating	23.58	2.83	0.00	0.00
Fissure/fistula/rupture	2.83	1.89	1.89	0.94
Flashbacks	5.66	74.53	56.60	20.75
Gender or body dysphoria	0.00	0.94	0.00	0.00
Genital warts	0.00	0.00	0.00	0.94
GI: reflux, gastric, digestive, diarrhoea	44.34	23.58	9.43	4.72
Gunshot wounds (GSW)	0.94	0.94	0.00	0.00
Guilt/Self-blame	39.62	42.45	39.62	23.58
Haemorrhoids	4.72	3.77	2.83	1.89
Hallucinations	2.83	1.89	0.00	0.94
Headaches/migraines	42.45	27.36	15.09	11.32
Hernia	0.00	2.83	3.77	1.89

SUPPLEMENTARY TABLE<br>**Symptom and Condition Prevalence over Time 1-4**				
**Symptoms**	**T1**	**T2**	**T3**	**T4**
Homicidal thoughts	0.00	1.89	3.77	2.83
Hopelessness	80.19	54.72	29.25	20.75
Hypersensitivity/hypervigilance	0.00	5.66	6.60	6.60
Hypertension/ heart disease	6.60	11.32	8.49	7.55
Impact on sexual relations; thoughts, identity	1.89	40.57	27.36	23.58
Impotence/erectile dysfunction	9.43	54.72	30.19	26.42
Inability to cry or feel emotions/numbness	2.83	7.55	1.89	2.83
Incontinence/leakage	21.70	18.87	10.38	7.55
Increased or unusual sexual desire	0.00	0.94	0.94	0.94
Infection	49.06	16.98	0.94	0.00
Infertility	0.00	1.89	1.89	1.89
Insomnia/sleep disturbance	94.34	91.51	66.04	38.68
Intrusive memories	5.66	88.68	80.19	68.87
Itching or irritation	72.64	53.77	7.55	5.66
Joining up to fight	0.00	22.64	8.49	2.83
Lack of appetite/anorexia	1.89	7.55	8.49	7.55
Lack of trust	1.89	59.43	53.77	66.98
Loss of confidence/ self-esteem/submissive/indecision	43.40	58.49	50.00	50.00
Loss of consciousness	62.26	4.72	0.94	0.00
Loss of digits	0.00	0.00	0.94	0.00
Loss of energy/deep tiredness/sleeping a lot	38.68	61.32	36.79	22.64
Loss of enjoyment/pleasure/joy	0.00	16.98	18.87	16.04
Loss of hair	6.60	3.77	3.77	1.89
Loss of nails (pulled or falling out)	8.49	2.83	0.00	0.94
Loss of sensation/numbness genitals	0.94	8.49	2.83	1.89
Loss of spacial or temporal awareness	26.42	14.15	0.00	0.94
Low mood/overly negative thoughts and assumptions about oneself/world	0.94	11.32	16.04	20.75
Low sexual desire	4.72	19.81	13.21	16.98
Maladaptive or risk-taking behaviours/disinhibition	4.72	8.49	0.94	1.89
Memory problems	29.25	40.57	26.42	12.26
Mood swings	0.00	4.72	5.66	9.43
Muscle atrophy	0.00	2.83	4.72	1.89
Nerve damage/trapped nerve	26.42	23.58	22.64	16.98
Nightmares	55.66	87.74	62.26	45.28
Numbness/apathy	9.43	5.66	3.77	4.72
Obsessive thoughts	0.00	4.72	9.43	7.55
Pain (chest/rib, abdomen, knees/legs, hand/arms, back)	100.00	94.34	78.30	64.15
Pain/burning during urination or defaecation	33.96	31.13	16.04	9.43
Pain/discomfort walking or sitting	31.13	24.53	14.15	14.15
Palpitations	27.36	3.77	3.77	2.83
Panic/panic attacks	31.13	21.70	11.32	5.66
Penile or testicular pain	1.89	2.83	0.00	0.94

**SUPPLEMENTARY TABLE**<br>**Symptom and Condition Prevalence over Time 1-4**				
**Symptoms**	**T1**	**T2**	**T3**	**T4**
Physical weakness	81.13	76.42	34.91	16.98
Pneumonia, TB, chest infections, etc.	4.72	1.89	0.00	0.00
Powerlessness/helplessness	78.30	49.06	22.64	16.98
Prolonged or intense fear	89.62	77.36	53.77	31.13
Prostate inflammation	0.94	0.94	0.94	1.89
Psychological "pain"	0.00	0.00	0.00	0.94
Restimulation/re-experience	0.94	54.72	19.81	9.43
Restricted movements	73.58	20.75	15.09	11.32
Sadness/crying, grief	88.68	83.96	59.43	48.11
Scar(s)	0.94	84.91	86.79	71.70
Self-isolation	0.00	75.00	58.00	65.00
Self-harm	12.26	0.94	0.00	1.89
Self-harm inflicted	0.00	0.94	0.94	0.94
Self-hate	0.00	0.00	0.00	3.77
Shame/humiliation	96.23	62.26	39.62	32.08
Skin infections or conditions	83.96	70.75	6.60	5.66
Skin tags	40.57	17.92	5.66	0.94
Sores/pustules	44.34	25.47	2.83	1.89
Speech difficulties	0.00	0.94	0.94	0.94
Startle reflex	27.36	70.75	39.62	25.47
Sexually transmitted disease/infections	0.94	3.77	0.94	0.00
Stretch marks	0.00	0.94	0.94	0.94
Substance misuse	0.00	1.89	1.89	2.83
Suicide attempts/ ideation of death	15.09	5.66	4.72	8.49
Swelling/inflammation	55.66	19.81	0.94	1.89
Tachycardia	6.60	1.89	0.94	0.94
Thoughts of revenge	19.81	33.02	14.15	3.77
Tinnitus or hearing difficulties	13.21	13.21	15.09	11.32
Urinary urgency or frequency	0.94	1.89	2.83	1.89
Urine retention	0.00	0.00	0.00	0.94
Urinary tract infections (UTIs)	3.77	10.38	5.66	7.55
Varicose veins	0.00	0.94	0.00	0.00
Vision issues	18.87	16.98	14.15	10.38
Vomiting/nausea	24.53	4.72	1.89	0.94
Weight gain	0.00	4.72	9.43	3.77
Weight loss; inadequate food/starvation	7.55	71.70	5.66	0.00
Wound/scar/bleeding of genitals/anus	16.04	14.15	8.49	6.60

Supplementary material: Association Between Types of Violence Experienced and Reported Sexual and Reproductive Health Conditions

While not among the ten most prevalent symptoms that were reported, participants also reported long-term impactful sexual and reproductive health (SRH) conditions, such as genital/anal wounds and scars, urinary incontinence, erectile dysfunction, and problems with sexual relations. Erectile dysfunction was commonly reported among participants, with 54·7% of men reporting this symptom during the post-detention period (Time 2), 30·2% of men at Time 3, and 26·4% of men at Time 4. Almost half (40·6%) reported difficulty with sexual relations at Time 2, followed by 27·3% at Time 3, and 23·6% at Time 4.

Based on relevant literature review and FME experience, we performed a preliminary analysis to test for the associations between the specific types of violence experienced and SRH symptoms. Our hypothesis was that there would be a significant association between direct genital violence or penetrative sexual violence and SRH symptoms at Time 4. **Table 2** shows the prevalence of SRH conditions among men who experienced specific types of violence at Time 4. While a history of direct trauma was associated with higher rates of SRH conditions, being forced to witness sexual violence and collective humiliation with sexualized elements was significant associated with painful urination and the presence of wounds in the genitals or anus, respectively.

Table 2. Prevalence of Reported Sexual and Reproductive Health Conditions at Time 4 Among Men Who Experienced Specific Types of Violence (n=106)

		**Type of Violence Experienced**					
		**Violence to genitals/anus (n=48)**	**Any form of rape (n=10)**	**Burns or electrocution of body part (n=51)**	**Forced to witness sexual violence (n=46)**	**Collective humiliation with sexualized elements (n=32)**	**Study population (n=106)**
**Reported Sexual and Reproductive Health Conditions**	**Erectile Dysfunction**	41·7%<br>P=0·003	70.0%<br>P=0·004	39·2%<br>P=0·007	NS	NS	26·4%
	**Impact on sexual relations, thoughts, and identity**	37·5%<br>P=0·005	60.0%<br>P=0·014	37·2%<br>P=0.003	NS	NS	23·6%
	**Pain and burning on urination**	18·8%<br>P=0·008	NS	NS	17·4%<br>P=0·034	NS	9·4%
	**Presence of wounds, scars or bleeding of genitals or anus**	14·6%<br>P=0·009	50.0%<br>P<0·001	NS	NS	15·6%<br>P=0·042	6·6%

Note: NS = Not Significant; df=degrees of freedom=1 for all

In the second analysis, we focused on men who presented with specific SRH symptoms at Time 4 to determine the prevalence of specific types of violence experienced within these symptom groups. **Table 3** presents the specific forms of violence that were present at a higher prevalence in sub-groups of men reporting different SRH symptoms at Time 4 when compared to the overall study population.

**Table 3. Rates of Specific Types of Violence Experienced Among Men Who Reported Specific Sexual and Reproductive Health Conditions at Time 4 (n=106)**						
		**Reported Sexual and Reproductive Health Conditions**				
		**Pain and burning on urination**	**Erectile dysfunction**	**Impact on sexual relations, thoughts, and identity**	**Presence of wounds, scars, or bleeding of genitals or anus**	**Study group n=106**
**Type of Violence Experienced**	**Violence to genitals/ anus**	90.0% P=0·008	71·4% P=0·007	NS	100% P=0·009	45·3%
	**Forced to witness sexual violence**	80.0% P=0·034	NS	NS	NS	43·4%
	**Any form of rape**	NS	25.0% P=0·004	24.0% P=0·014	71·4% P<0·001	9·4%
	**Burning or electrocution of body part of their body**	NS	71·4% P=0·003	76.0% P=0·003	NS	48·1%
	**Collective humiliation with a sexualized element**	NS	NS	NS	71·4% P=0·042	??

Note: NS = Not Significant; df=degrees of freedom=1 for all

These analyses represent initial findings which warrant further investigation. The exact relationship between the specific types of violence and the subsequent development of SRH conditions needs further elucidation.
